# Audit for Patients Who Are Discharged on Community Treatment order (CTO) in North East Part of Essex: Exploring the Section Paperwork and the Readmissions in 2 Year Period

**DOI:** 10.1192/bjo.2024.525

**Published:** 2024-08-01

**Authors:** Bushra Ahmed, Hem Raj Pal, Rashed Alhadi

**Affiliations:** 1 Essex Partnership NHS Foundation Trust, Colchester, United Kingdom; 2 Essex Partnership NHS Foundation Trust, Colchester, United Kingdom; 3 NHS Tayside Foundation Trust, Dundee, Scotland, United Kingdom

## Abstract

**Aims:**

- To find out discrepancies around community treatment order section paperwork and renewal hearings.
- Explore if CTO is helpful in delaying relapse of mental illness.

**Methods:**

All inpatients from general adult male and female wards in the North East Essex area in the last 3–5 years who were detained under section 3 of the Mental Health Act and were discharged on a Community treatment order were included. All included patients were followed up for a period of two years and data was reviewed to know if the standard guidelines regarding the CTO paperwork completion and renewal hearings were followed.

Data about episodes of further recalls to hospital, further revocation or discharges on CTO during that two year period for these patients is included.

Information about the timely filling of the CTO forms and uploading on the system upon readmission is explored wherever applicable.

Finally, the time duration between discharges and each readmission is explored.

**Results:**

Total no. of patients: 13, Male: 10, Female: 3

Out of the 13 patients,

One had 4 readmissions in the consequent two year period (Days since last discharge – 158, 80, 14, 365 days), duration of each admission: (39, 9, 71, 53) days.

Two had 2 readmissions (on days 623 and 80: On day 65 and 9), Duration of each stay (6 and 90 days; 80 and 164 days).

Four patients had 1 readmission (on days 683, 133, 30, 723) and duration of stay is (14, 33, 1510 and 1460 days).

Six patients never had any admission.

As for the tribunal hearing, one patient's tribunal hearing was missed, one of them did not attend, one had his CTO rescinded and one was admitted soon after. Rest of the patients had their regular timely hearings and were regularly reviewed in the community.

Out of 13, only 3 patients had appealed against the CTO, had tribunal hearing.

Out of 13 patients, only one patient had his CTO lapsed and he had two readmissions during the 2 years follow up.

Delay in admission following recall was due to section 135 being issued.

CTO3 paperwork was missing in two cases.

A second CTO3 or recall notice was issued in 4 cases, in 1 case, reason was not documented, in 2 cases, patient agreed for informal stay but later did not comply with care plan. In one case, reason was not documented.

**Conclusion:**

CTO paperwork are missed in rare cases and could be avoided by reminders from Mental Health Act Office.

CTO renewal hearings take place regularly as per mental health act guidelines, though in rare cases, meeting is missed. This could be avoided by having patients discharged on CTO to be booked for timely reviews beforehand.

The audit is too small and is inconclusive to indicate if CTOs prevent readmissions and relapses and hence future study with more sample size is called for.

